# Fetal and neonatal dioxin exposure causes sex-specific metabolic alterations in mice

**DOI:** 10.1093/toxsci/kfad042

**Published:** 2023-04-28

**Authors:** Myriam P Hoyeck, Rayanna C Merhi, Cameron Tulloch, Kaitlyn McCormick, Shahen Mohammed Abu Hossain, Antonio A Hanson, Jennifer E Bruin

**Affiliations:** Department of Biology, Carleton University, Ottawa, Ontario K1S 5B6, Canada; Institute of Biochemistry, Carleton University, Ottawa, Ontario K1S 5B6, Canada; Department of Biology, Carleton University, Ottawa, Ontario K1S 5B6, Canada; Institute of Biochemistry, Carleton University, Ottawa, Ontario K1S 5B6, Canada; Department of Biology, Carleton University, Ottawa, Ontario K1S 5B6, Canada; Institute of Biochemistry, Carleton University, Ottawa, Ontario K1S 5B6, Canada; Department of Biology, Carleton University, Ottawa, Ontario K1S 5B6, Canada; Institute of Biochemistry, Carleton University, Ottawa, Ontario K1S 5B6, Canada; Department of Biology, Carleton University, Ottawa, Ontario K1S 5B6, Canada; Institute of Biochemistry, Carleton University, Ottawa, Ontario K1S 5B6, Canada; Department of Biology, Carleton University, Ottawa, Ontario K1S 5B6, Canada; Institute of Biochemistry, Carleton University, Ottawa, Ontario K1S 5B6, Canada; Department of Biology, Carleton University, Ottawa, Ontario K1S 5B6, Canada; Institute of Biochemistry, Carleton University, Ottawa, Ontario K1S 5B6, Canada

**Keywords:** β-cell mass, hypoglycemia, diabetes, dioxin, early-life pollutant exposure, metabolic adaptability

## Abstract

Epidemiological studies report associations between early-life exposure to persistent organic pollutants (POPs) and impaired metabolic homeostasis in adulthood. We investigated the impact of early-life exposure to low-dose 2,3,7,8-tetrachlorodibenzo-*p*-dioxin (TCDD or ‘dioxin’) on the establishment of β-cell area during the perinatal period, as well as β-cell health and glucose homeostasis later in life. Adult female mice were injected with either corn oil (CO; vehicle control) or TCDD (20 ng/kg/day) 2×/week throughout mating, pregnancy, and lactation; offspring were thus indirectly exposed to maternal TCDD *in utero* and during lactation, with pollutant exposure ending at weaning. All offspring were maintained on chow diet from weaning until 12–17 weeks of age, after which a subset of CO- and TCDD-exposed offspring were transferred to a 45% high fat diet (HFD) as a metabolic stressor for an additional 10 weeks. TCDD significantly upregulated cytochrome P450 1a1 (*Cyp1a1*) gene expression in offspring pancreas at birth and weaning, indicating that maternal TCDD directly reaches the developing pancreas. TCDD-exposed pups were transiently hypoglycemic at birth and females were born with reduced % β-cell area, which persisted into adulthood. Early-life TCDD exposure had no persistent long-term effects on glucose homeostasis in chow-fed offspring, but when transferred to HFD, TCDD-exposed female offspring had a delayed onset of HFD-induced hyperglycemia, more pronounced HFD-induced hyperinsulinemia, and increase % PCNA^+^ β-cells compared with CO-exposed female offspring. This study demonstrates that early-life exposure of mice to TCDD has modest effects on metabolic health in chow-fed offspring but alters metabolic adaptability to HFD feeding in females.

Worldwide prevalence of youth- and adult-onset diabetes has drastically increased over recent decades, and is projected to further increase 2- to 4-fold by 2050 (Imperatore *et al.*, 2012; IDF, 2021). This rapid rise in diabetes rates cannot be explained by genetic predisposition alone, meaning that environmental factors are also influencing diabetes pathogenesis. Epidemiological studies consistently report associations between exposure to persistent organic pollutants (POPs) and increased diabetes risk or incidence in adults (Hoyeck *et al.*, 2022; Lee *et al.*, 2014; Sun *et al.*, 2017; Taylor *et al.*, 2013; Thayer *et al.*, 2012). Early-life exposure to POPs has also been linked to hyperglycemia (Baumert *et al.*, 2022), increased metabolic syndrome (Warner *et al.*, 2014; 2017), hypoinsulinemia (Jensen *et al.*, 2014; Park *et al.*, 2016), reduced HOMA-β (Jensen *et al.*, 2014; Park *et al.*, 2016), and higher non-fasted insulin levels (Tang-Péronard *et al.*, 2015) in children. These studies suggest that early-life POP exposure increases the risk of developing β-cell dysfunction and hyperglycemia later in life, but this association needs to be confirmed in a controlled model system.

POPs are a diverse class of lipophilic environmental contaminants that resist degradation, leading to widespread global dispersion and bioaccumulation (Islam *et al.*, 2018; Jamieson *et al.*, 2017; Martin *et al.*, 2004). Dioxins and dioxin-like compounds are a broad subclass of POPs that are formed as by-products of combustion processes (Kanan and Samara, 2018). The biological effects of dioxins are largely mediated through activation of the aryl hydrocarbon receptor (AhR), and AhR-target genes, including cytochrome P450 (*Cyp*)*1a1*; AhR activation can generate reactive oxygen species, impair Ca^2+^ signaling, and alter cell survival (Hoyeck *et al.*, 2022; Sayed *et al.*, 2022; Sorg, 2014). AhR has also been reported to play an important role in organ development (eg, heart, liver, spleen, skin) and maintenance of glucose and lipid homeostasis (Sayed *et al.*, 2022).

Our lab has shown that systemic exposure to the highly persistent dioxin, 2,3,7,8-tetrachlorodiobenzo-*p*-dioxin (TCDD, aka ‘dioxin’), induced *Cyp1a1* expression in adult mouse islets (Hoyeck *et al.*, 2020a; Ibrahim *et al.*, 2020; Matteo *et al.*, 2021), confirming that the endocrine pancreas is directly exposed to POPs *in vivo*. We also showed that prolonged low-dose TCDD exposure (20 ng/kg/day, 2×/week) accelerated high-fat diet (HFD)-induced hyperglycemia and impaired glucose-induced plasma insulin levels in adult female but not male mice (Matteo *et al.*, 2021). Transient low-dose TCDD exposure in female mice throughout pregnancy and lactation also increased susceptibility to HFD-induced obesity and dysglycemia postpartum (Hoyeck *et al.*, 2020b). These TCDD-exposed dams displayed reduced islet size, decreased MAFA^+^ β-cells, and increased cytoplasmic proinsulin accumulation compared with CO-exposed dams (Hoyeck *et al.*, 2020b). Collectively, these data suggest that low-dose dioxin exposure has long-lasting adverse effects on glucose homeostasis and β-cell health in mice.

Dioxins cross the placenta (Li *et al.*, 1995; Weber and Birnbaum, 1985) and are detectable in breast milk (Aerts *et al.*, 2019), thus offspring exposure begins *in utero*. Embryogenesis and the early neonatal period are critical windows for β-cell development. β-cell mass is largely established by weaning (ie, 3 weeks of age) in mice (Baeyens *et al.*, 2018; Dhawan *et al.*, 2007; Gregg *et al.*, 2012; Salinno *et al.*, 2019) and by ∼2 years of age in humans (Gregg *et al.*, 2012; Meier *et al.*, 2008). Beyond this early developmental window, there is limited capacity for β-cell growth and regeneration (Dhawan *et al.*, 2007; Elsakr and Gannon, 2017; Zhong and Jiang, 2019). Furthermore, β-cell maturation in mice occurs postnatally and is complete by weaning (Benitez *et al.*, 2012; Nair and Hebrok, 2015; Salinno *et al.*, 2019), whereas β-cell maturation in humans occurs during the last trimester of pregnancy and is largely complete before birth (Basile *et al.*, 2019; Nair and Hebrok, 2015). As such, early-life stressors that disrupt β-cell differentiation and/or maturation can lead to a life-long susceptibility to developing diabetes (Inadera, 2013; Jiang *et al.*, 2013; Nielsen *et al.*, 2014; Remacle *et al.*, 2007).

We previously reported long-term adverse metabolic health outcomes in dams following TCDD exposure during pregnancy and lactation (Hoyeck *et al.*, 2020b), but here we focus on the health outcomes of their offspring. We investigated the impact of transient low-dose exposure to TCDD, via maternal administration during fetal and neonatal development, on islet composition and β-cell health at birth and weaning in male and female offspring. TCDD exposure stopped when offspring were weaned at 3 weeks of age, but we continued to assess offspring metabolic health until postnatal weeks 22–27, including how offspring adapted to a HFD transition later in life. We report that early-life exposure to TCDD caused hypoglycemia in male and female offspring at birth, an effect that persisted until weaning in male offspring. TCDD-exposed female offspring were also born with a reduced % β-cell area, which persisted until 27 weeks of age. Early-life TCDD exposure had no long-term effects on glucose homeostasis in chow-fed male or female offspring, but delayed the onset of HFD-induced hyperglycemia, caused more pronounced HFD-induced hyperinsulinemia and increased % fat mass in female offspring.

## Materials and methods

###  

#### Animals

All mice received *ad libitum* access to standard rodent chow (Harlan Laboratories, Teklad Diet No. 2018, Madison, WI) and were maintained on a 12-h light/dark cycle throughout the study. All experiments were approved by the Carleton University and University of Ottawa Animal Care Committees and carried out in accordance with the Canadian Council on Animal Care guidelines. Prior to beginning experimental protocols, animals were randomly assigned to treatment groups and matched for body weight and blood glucose to ensure that these variables were consistent between groups.

##### Cohort 1

**Figure 1.**
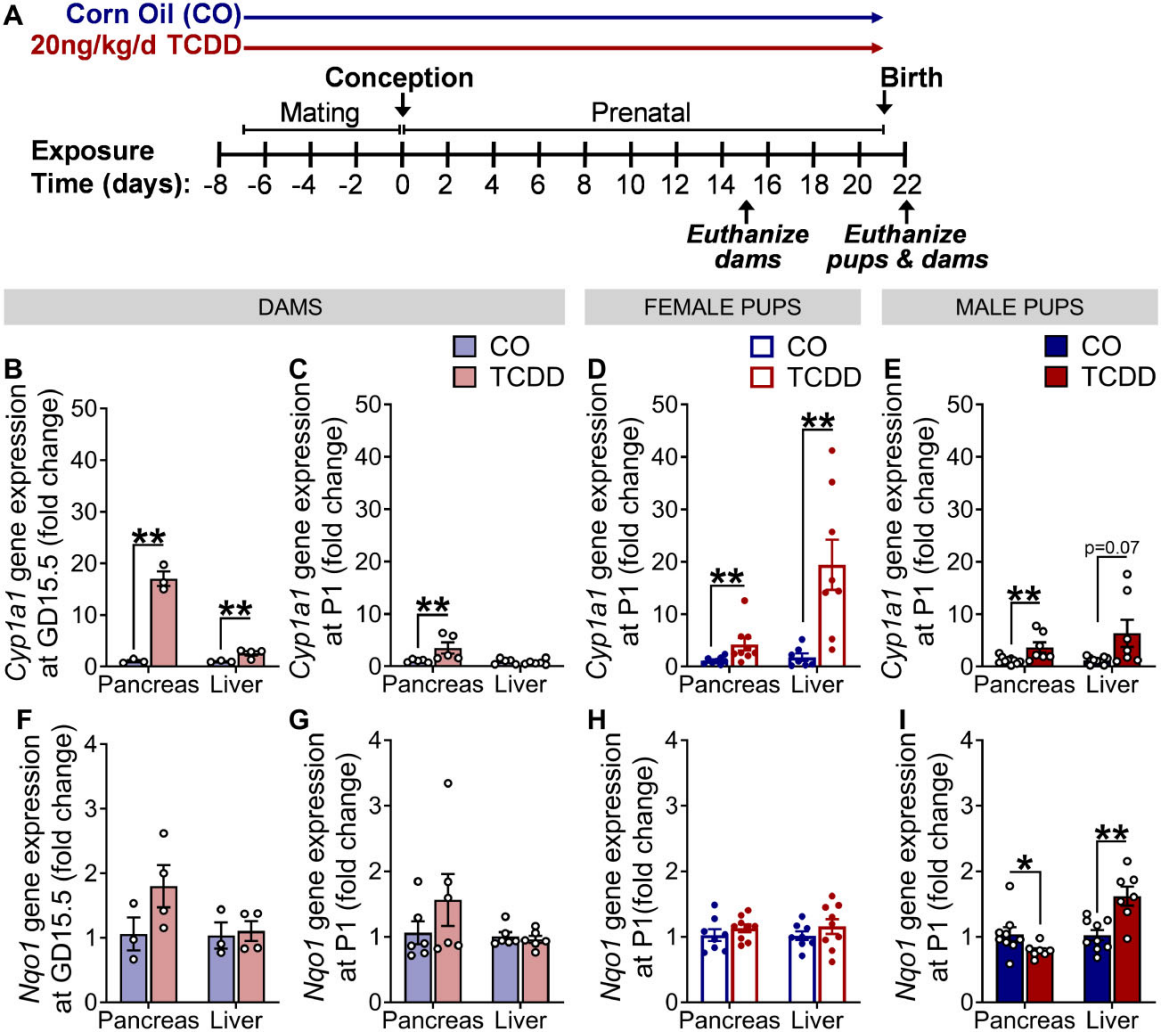
Low-dose TCDD exposure during pregnancy induced *Cyp1a1* expression in dam and offspring pancreas. (A) Schematic summary timeline of the study. Female mice were injected with either corn oil (CO) or 20 ng/kg/day TCDD 2×/week during mating and pregnancy (*n* = 6/group); offspring were indirectly exposed *in utero*. Dams were euthanized at gestational day (GD) 15.5 or postnatal day 1 (P1). Offspring were euthanized at P1. (B–E) *Cyp1a1* and (F–I) *Nqo1* gene expression were measured at (B and F) GD15.5 (*n* = 3–4/experimental group) and (C–E and G–I) P1 in (B, C, F, and G) dams (*n* = 5–6/group) and (D, E, H, and I) offspring (*n* = 1–2 pups/sex/litter, *n* = 5–6 different litters/group). All data is presented as mean±SEM. *p < .05 and **p < .01 versus CO. The following statistical tests were used: (B and H) two-tailed unpaired *t* test; (C, F, and G) two-tailed unpaired *t* test for pancreas, Mann–Whitney test for liver; (D and E) Mann–Whitney test; and (I) Mann–Whitney *t* test for pancreas, two-tailed unpaired test for liver.

Female and male C57BL/6 mice, 6- to 7-week old, were purchased from Charles River (Raleigh, NC) and acclimated for a week prior to starting experiments. Female mice (*n* = 6/group) received subcutaneous (s.c.) injections of corn oil (CO; 25 ml/kg, vehicle control; Sigma-Aldrich, No. C8267-2.5L, St Louise, MO) or a low-dose of TCDD (20 ng/kg/day; Sigma Aldrich, No. 48599) 2×/week starting 1 week prior to pairing with male mice and lasting throughout mating and pregnancy, as previously described (Hoyeck *et al.*, 2020b). As outlined in Figure 1A, offspring were indirectly exposed to TCDD *in utero* via their dams. The TCDD dose was selected based on studies showing that a similar dosing protocol induced *Cyp1a1* mRNA and enzyme activity in liver/lung tissues and produced an environmentally relevant steady-state tissue burden without overt TCDD toxicity (eg, weight loss, hepatic toxicity) (Bell *et al.*, 2007; NIo, 2006). Additionally, our lab has shown that this dose of TCDD significantly induced *Cyp1a1* in mouse islets and liver 2 weeks post-exposure (Ibrahim *et al.*, 2020), and was generally well-tolerated in mice following 7 and 12 weeks of prolonged exposure (Hoyeck *et al.*, 2020b; Matteo *et al.*, 2021).

**Figure 2.**
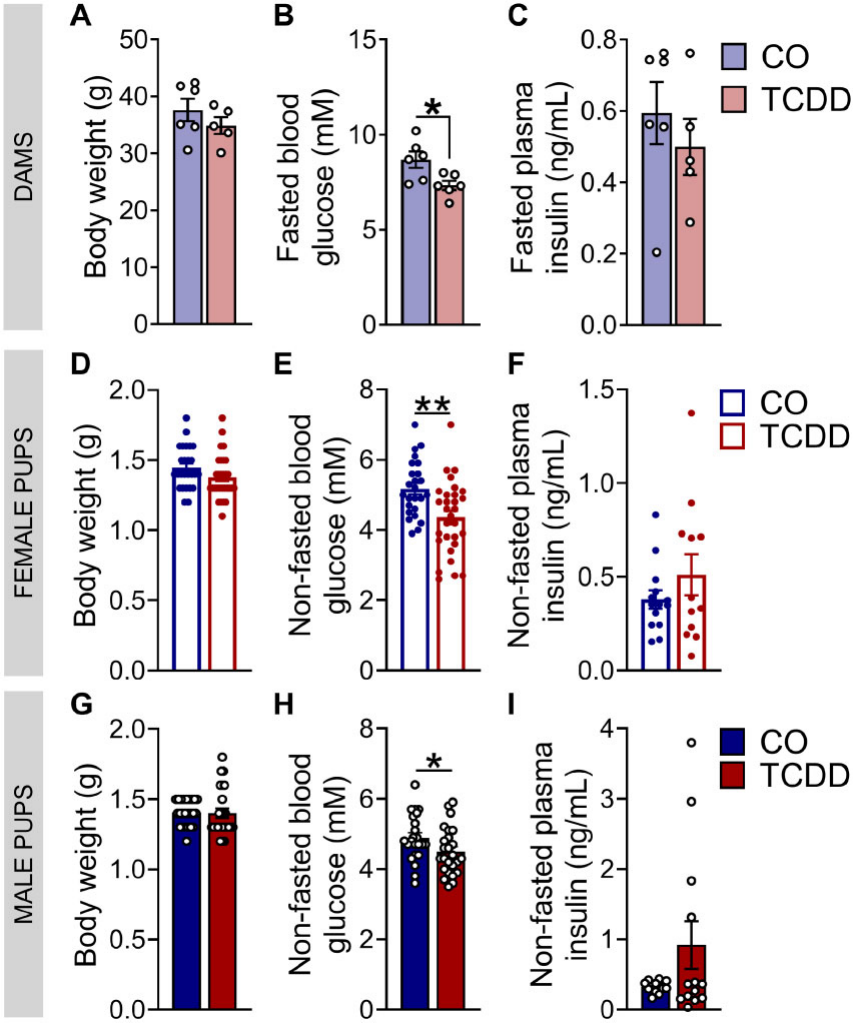
Low-dose TCDD exposure during pregnancy caused hypoglycemia in dams and offspring at postnatal day 1. (A) Body weight at ∼GD15.5, (B) fasted blood glucose at P1, and (C) fasted plasma insulin at P1 in dams. (D and G) Body weight, (E and H) non-fasted blood glucose, and (F and I) non-fasted plasma insulin in offspring at P1, with each dot representing an individual pup (*n* = 1–9 pups/sex/litter, *n* = 5–6 different litters/group). All data are presented as mean±SEM. *p < .05 and **p < .01 versus CO. The following statistical tests were used: (A–C, E, F, and H) two-tailed unpaired *t* test; (D, G, and I) Mann–Whitney test.

**Figure 3.**
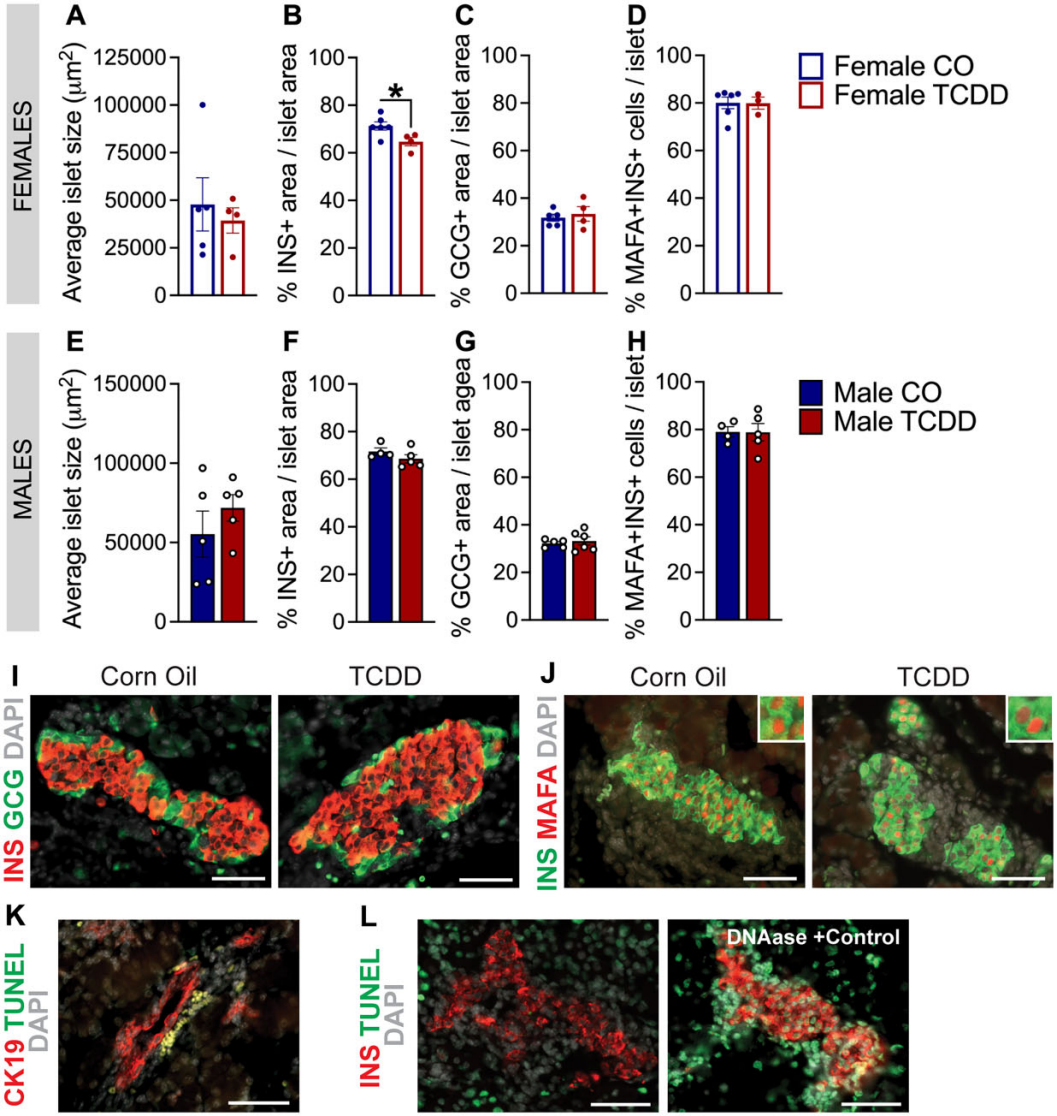
Low-dose TCDD exposure *in utero* reduced % β-cell area per islet in female but not male offspring at P1. (A and E) Average islet size, (B and F) % insulin (INS)^+^ area/islet area, (C and G) % glucagon (GCG)^+^ area/islet area, and (D and H) % MAFA^+^INS^+^ cells/islet in (A–D) female offspring (*n* = 1 pup/litter, *n* = 3–6 different litters/group) and (E–H) male offspring (*n* = 1 pup/litter, *n* = 4–6 different litters/group). Representative images showing immunofluorescence staining for (I) insulin and glucagon, (J) insulin and MAFA, (K) CK19 and TUNEL, and (L) insulin and TUNEL, including a pancreas section treated with DNase as a positive control. Scale bars = 50 µm. All data are presented as mean±SEM. Individual data points on bar graphs represent biological replicates (ie, different litters); a maximum of 1 male and 1 female pup per litter was used for this analysis. *p < .05 versus vehicle CO. The following statistical tests were used: (A–C and E–H) two-tailed unpaired *t* test; (D) two-tailed Mann–Whitney test.

All dams and their pups were euthanized on postnatal day 1 (P1). Pancreas and liver were harvested from all dams and stored in RNAlater for qPCR. Within a litter, pancreas and liver were harvested from different subsets of pups for storage in either RNAlater (*n* = 1–2 pups/sex/litter) or 4% paraformaldehyde (PFA) for 24 h, followed by long-term storage in 70% ethanol (*n* = 1 pup/sex/litter) (Figs. 1–3).

##### Cohort 2

**Figure 4.**
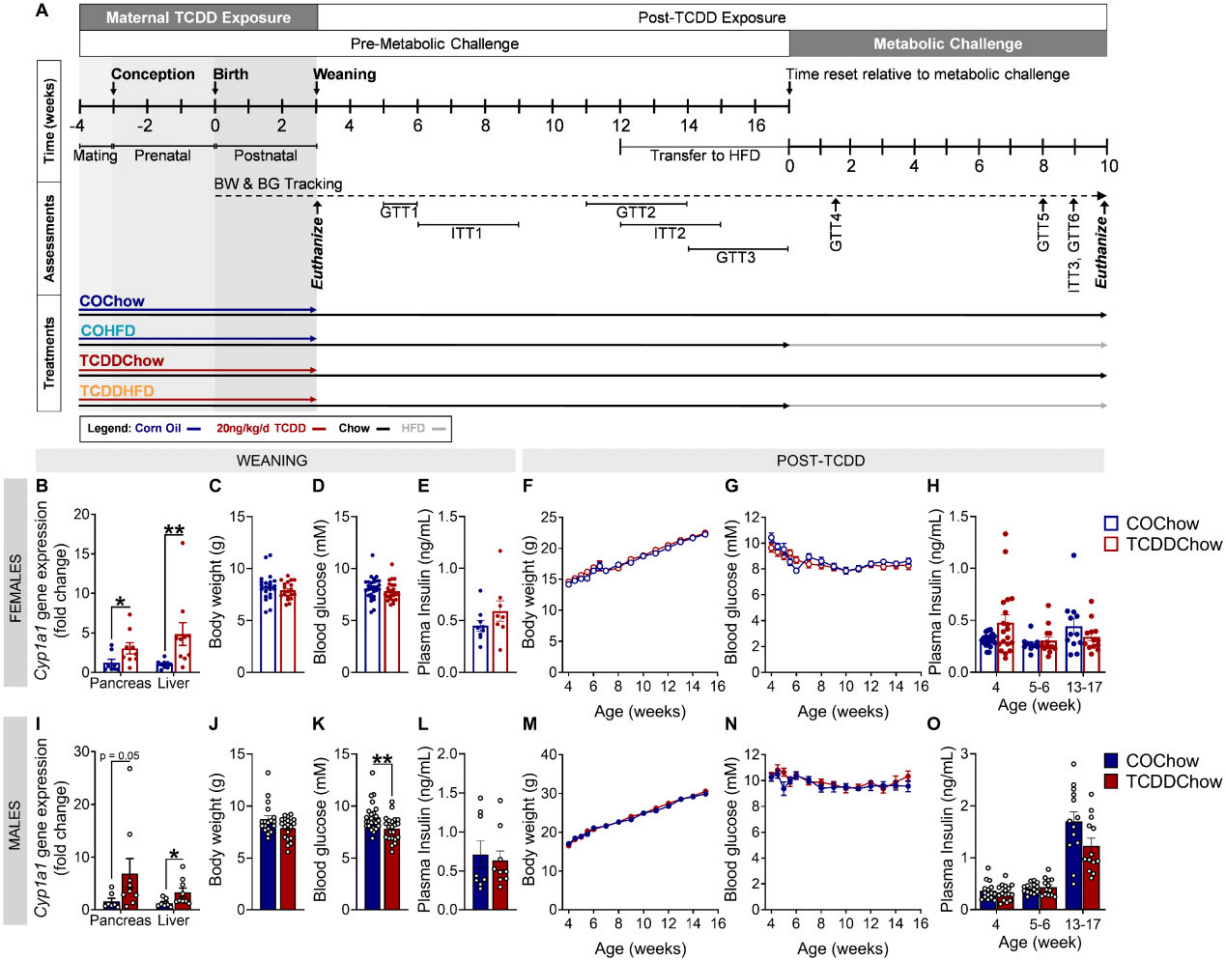
TCDD-exposed male offspring were transiently hypoglycemic at weaning, but no persistent effects on body weight, blood glucose, or plasma insulin levels were observed in either sex. (A) Schematic summary timeline of the study. A new cohort of female mice were injected with either corn oil (CO) or 20 ng/kg/day TCDD 2×/week during mating, pregnancy, and lactation; offspring were indirectly exposed *in utero* and postnatally during lactation up until weaning (‘Maternal TCDD Exposure’ window). Male and female offspring were maintained on chow diet until postnatal weeks 12–17 (9–14 weeks following the last TCDD exposure; ‘Post TCDD Exposure’ window). This age range results from the duration of mating, with a 5-week difference between the first and last litters. At postnatal weeks 12–17, a subset of CO- and TCDD-exposed offspring were transferred to a 45% HFD or remained on chow diet for an additional 10 weeks. BW = body weight, BG = blood glucose, GTT = glucose tolerance test, ITT = insulin tolerance test. (B and I) *Cyp1a1* gene expression in pancreas and liver of (B) female and (I) male offspring at weaning (*n* = 1–2/sex/litter, *n* = 9–10 different litters/group). (C and J) Body weight, (D and K) non-fasted blood glucose, and (E and L) non-fasted plasma insulin levels were measured at postnatal week 3 in (C–E) female and (J–L) male offspring (*n* = 1–3/sex/litter, *n* = 8–10 different litters/group). (F and M) Body weight and (G and N) fasting blood glucose were measured weekly between 4 and 16 weeks of age in (F and G) female and (M and N) male offspring (*n* = 1–3/sex/litter, *n* = 10 different litters/group). Fasted plasma insulin levels were measured at postnatal weeks 4, 5–6, and 13–17 in (H) females and (O) males (*n* = 1–3/sex/litter, *n* = 9–10 different litters/group). All data are presented as mean±SEM. Individual data points on bar graphs represent biological replicates (different mice). *p < .05 and **p < .01 versus CO. The following statistical tests were used: (B, C, and H–O) two-tailed Mann–Whitney test; (F, G, M, N) two-way REML ANOVA with Sidak’s test; and (D and E) two-tailed unpaired *t* test.

Female, 5-week old, and male, 7- to 8-week old, mice were purchased from Charles River (Raleigh, NC) and acclimated for a week prior to starting experiments. Female mice received s.c. injections of CO (25 ml/kg, *n* = 10) or a low-dose of TCDD (20 ng/kg/day; *n* = 10) 2×/week starting 1 week prior to pairing with male mice and lasting throughout mating, pregnancy, and lactation, as previously described (Hoyeck *et al.*, 2020b). As outlined in Figure 4A, offspring were exposed to maternal TCDD *in utero* and during lactation, with the last exposure at weaning (‘Maternal TCDD Exposure’ window). At P1, litters were culled to 6 pups/litter (*n* = 3/sex, except for 2 litters that had 3–5 females and 1 male) to ensure that litter size did not alter metabolic outcomes in our study. All offspring were maintained on chow diet (Harlan Laboratories, Teklad Diet, No. 2018) from weaning until postnatal week 12–17 (‘Post-TCDD’ window); this range reflects differences in the duration of mating, with a 5-week difference between the first and last pregnancies. A subset of CO- and TCDD-exposed offspring were transferred to a 45% HFD (Research Diets, No. D12451, New Brunswick, NJ) or remained on chow for an additional 10 weeks (‘Metabolic Challenge’ window), generating the following experimental groups (*n* = 1–2/sex/litter/group): COChow, COHFD, TCDDChow, and TCDDHFD (see Supplementary Figure 1 for additional information about sample sizes).

**Figure 5.**
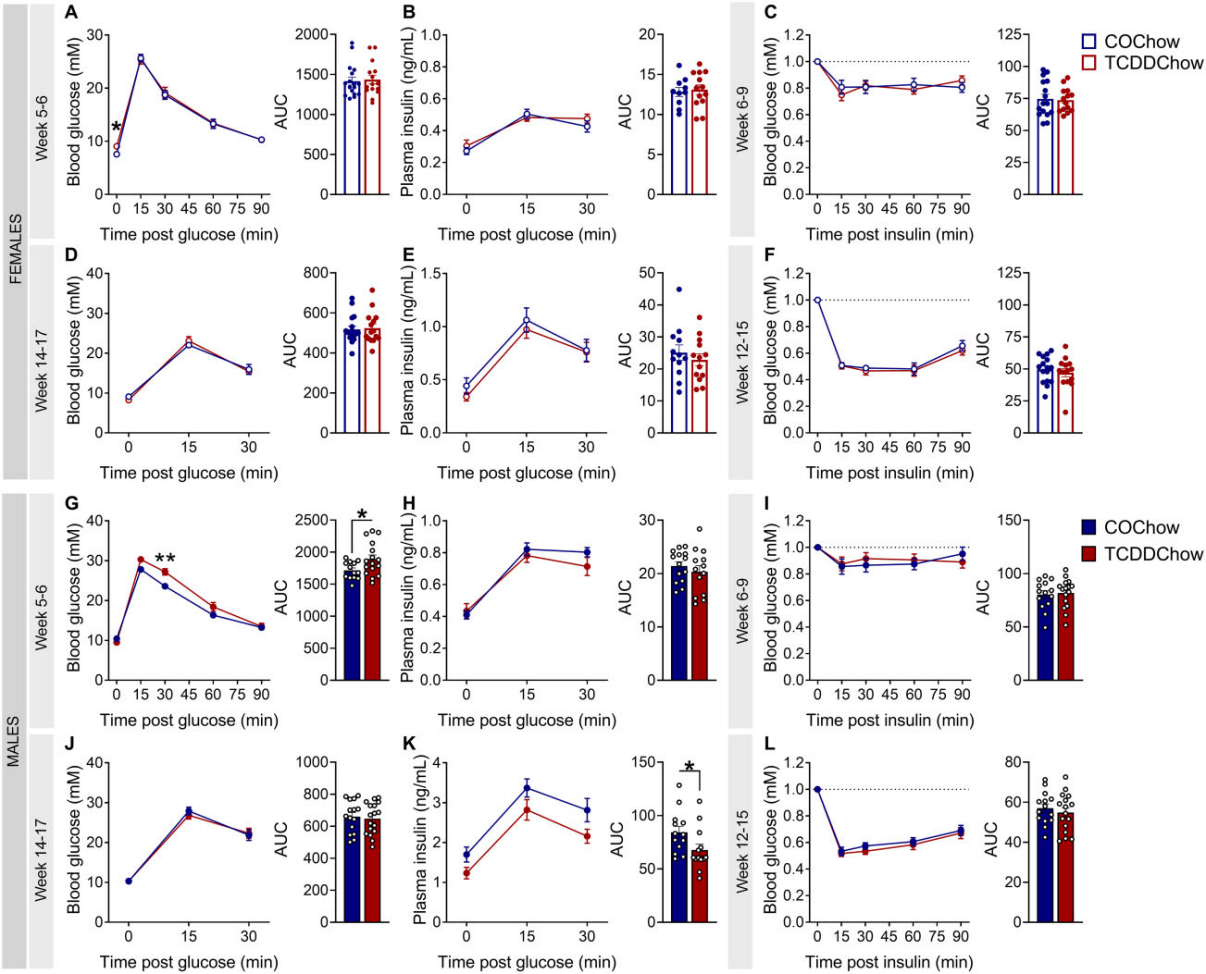
TCDD-exposed male offspring showed transient glucose intolerance and reduced glucose-stimulated plasma insulin levels. Glucose tolerance tests (GTTs) and insulin tolerance tests (ITTs) were performed at postnatal week 5–9 (ie, 2–6 weeks post-TCDD) and postnatal week 12–17 (ie, 9–14 weeks post-TCDD) (see Figure 4A for study timeline) (*n* = 1–3/sex/litter, *n* = 9–10 different litters/group). (A, D, G, and J) Blood glucose and (B, E, H, and K) plasma insulin levels during GTTs at (A, B, G, and H) postnatal week 5–6 and (D, E, J, and K) postnatal week 14–17 in (A, B, D, and E) female and (G, H, J, and K) male offspring. Blood glucose levels during ITTs at (C and I) postnatal weeks 6–9 and (F and L) postnatal weeks 12–15 in (C and F) female and (I and L) male offspring. ITT values are normalized relative to time zero for each mouse. All data are presented as a line graph and area under the curve. All data are presented as mean±SEM. Individual data points in bar graphs represent biological replicates (different mice). *p < .05 and **p < .01. The following statistical tests were used: (A) line graph, two-way RM ANOVA with Sidak’s test; bar graph, two-tailed Mann–Whitney test; (B–L), line graph, two-way RM ANOVA with Sidak’s test; bar graph, two-tailed unpaired *t* test.

**Figure 6.**
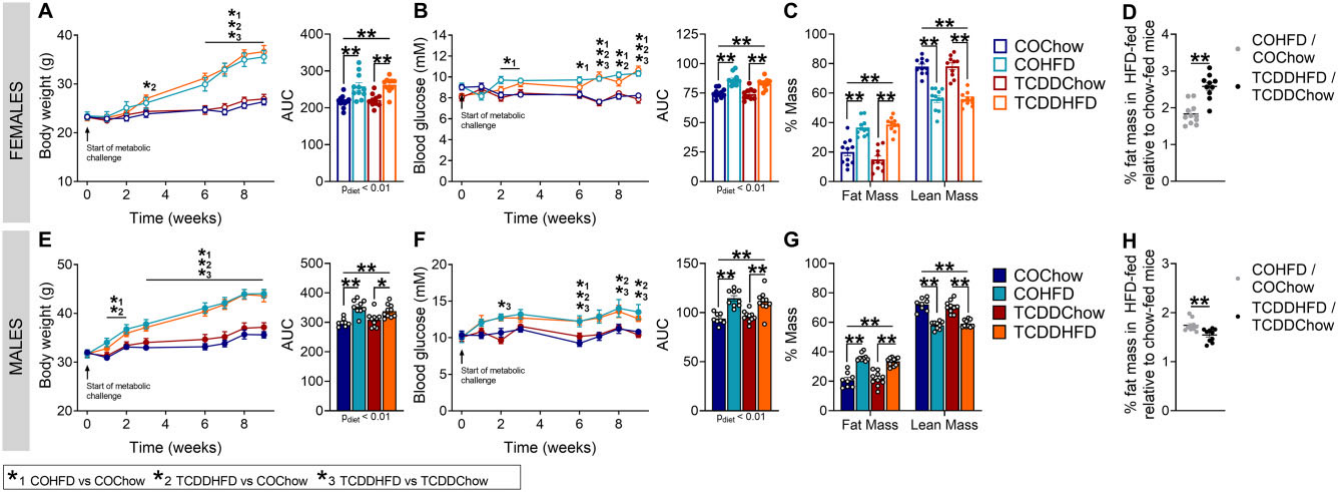
Early-life TCDD exposure increased % fat mass in HFD-fed females but decreased % fat mass in HFD-fed males relative to chow. At postnatal weeks 12–17 (ie, 9–14 weeks post-TCDD), a subset of CO- and TCDD-exposed offspring were transferred to 45% HFD feeding or remained on standard chow for another 10 weeks (‘Metabolic Challenge’; see Figure 4A for study timeline) (*n* = 1–2/sex/litter, *n* = 8–10 different litters/group). (A and E) Body weight and (B and F) fasting blood glucose were measured weekly in (A and B) female and (E and F) male offspring; data are presented as a line graph and area under the curve. Fat mass and lean mass were measured by EchoMRI after 9 weeks of HFD feeding in (C) female and (G) male offspring. % fat mass in HFD-fed offspring normalized to their chow-fed counterpart in (D) female and (H) male offspring. All data are presented as mean±SEM. Individual data points in bar graphs represent biological replicates (different mice). *p < .05 and **p < .01. The following statistical tests were used: A, line graph, two-way RM ANOVA with Tukey’s multiple comparison test; bar graph, two-way ANOVA with Tukey’s multiple comparison test; (B, E, and F) line graph, two-way REML ANOVA with Tukey’s multiple comparison test; bar graph, two-way ANOVA with Tukey’s multiple comparison test; (C and G) two-way ANOVA with Tukey’s multiple comparison test; (D and H) two-tailed unpaired *t* test. The following comparison groups were used for statistical analysis: 1, COHFD versus COChow; 2, TCDDHFD versus COChow; 3, TCDDHFD versus TCDDChow.

**Figure 7.**
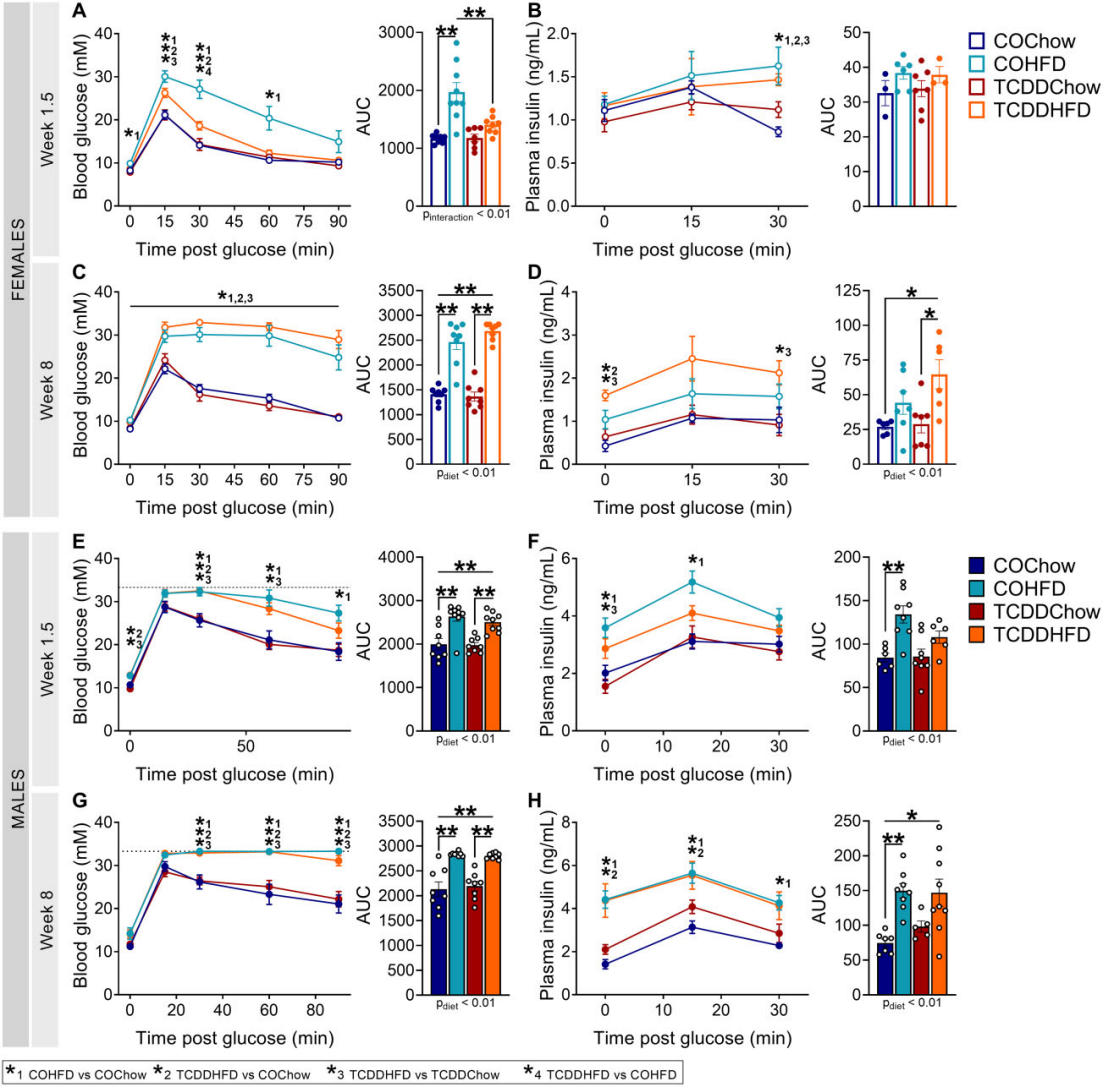
Early-life TCDD exposure delayed the onset of hyperglycemia and caused more pronounced hyperinsulinemia in HFD-fed female offspring. Glucose tolerance tests (GTTs) were performed after 1.5 and 8 weeks of HFD feeding (see Figure 4A for study timeline) (*n* = 1–2/sex/litter, *n* = 8–10 different litters/group). (A, C, E, and G) Blood glucose and (B, D, F, and H) plasma insulin levels at (A, B, E, and F) week 1.5 and (C, D, G, and H) week 8 of HFD feeding in (A–D) female and (E–H) male offspring; data are presented as a line graph and area under the curve. All data are presented as mean±SEM. Individual data points in bar graphs represent biological replicates (different mice). *p < .05 and **p < .01. (A–H) Line graph, two-way REML ANOVA with Tukey’s multiple comparison test; bar graph, two-way ANOVA with Tukey’s multiple comparison test. The following comparison groups were used for statistical analysis: 1, COHFD versus COChow; 2, TCDDHFD versus COChow; 3, TCDDHFD versus TCDDChow; 4, TCDDHFD versus COHFD.

**Figure 8.**
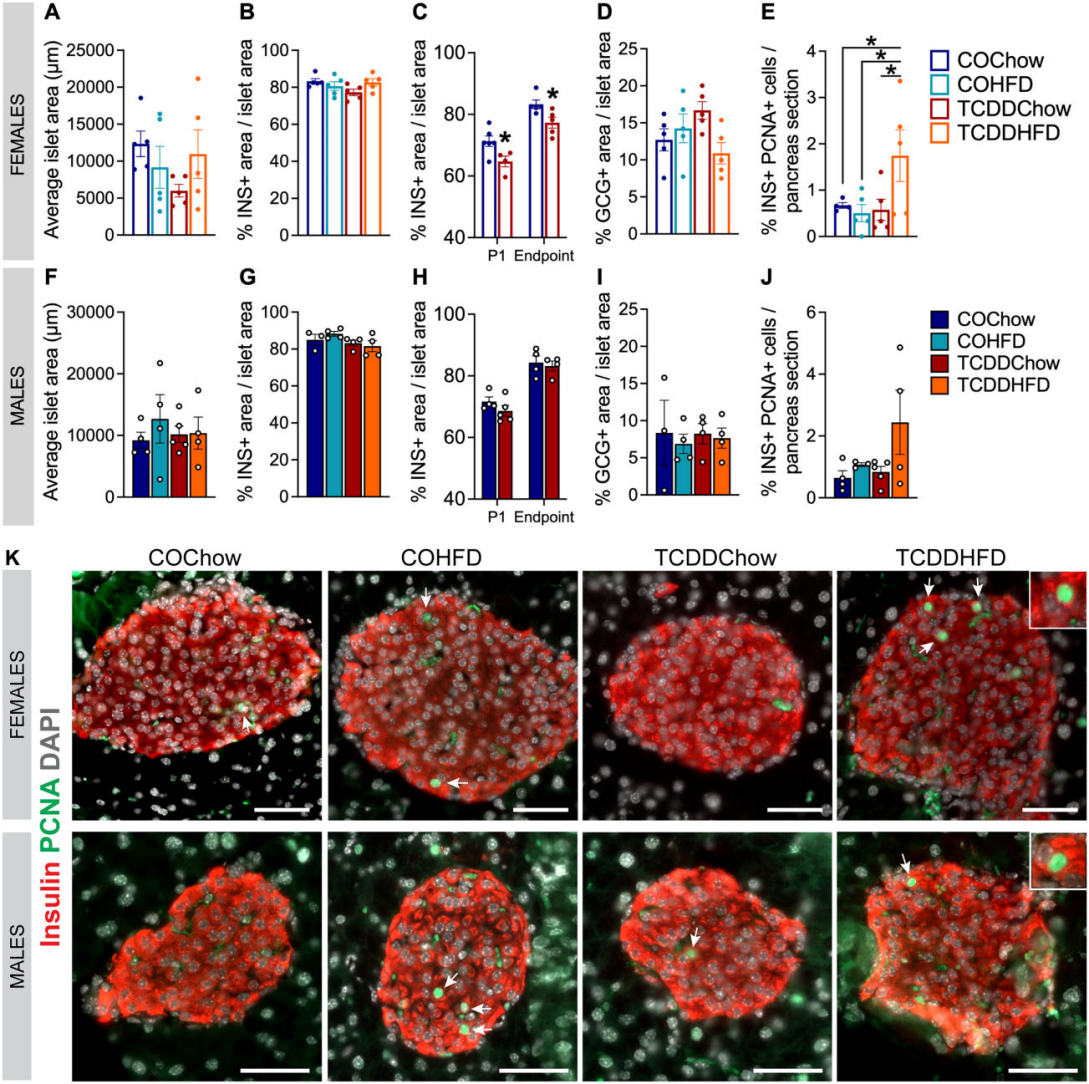
TCDDChow female offspring continued to have reduced % β-cell area into adulthood and TCDDHFD females had increased % PCNA^+^ β cells. Pancreas was harvested from offspring at 10 weeks post-HFD for histological analysis (see Figure 4A for study timeline) (*n* = 1/sex/litter, *n* = 4–5 different litters/group). (A and F) Average islet size, (B, C, G, H) % insulin (INS)^+^ area/islet area, (D and I) % glucagon (GCG)^+^ area/islet area, and (E and J) % insulin^+^ PCNA^+^ cells/pancreas section. (C and H) % insulin (INS)^+^ area/islet area at P1 and endpoint presented side by side. (I) Representative images showing immunofluorescent staining for insulin and PCNA. Scale bars = 50 μm. All data are presented as mean±SEM. Individual data points in bar graphs represent biological replicates (different mice). The following statistical tests were used: (A, B, D–G, I, and J) two-way ANOVA with Tukey’s multiple comparison test; (C) two-tailed unpaired *t* test at P1; two-tailed Mann–Whitney test at endpoint; and (H) two-tailed unpaired *t* test at P1 and endpoint.

At approximately day 15.5 of pregnancy (gestational day, GD15.5), pancreas and liver were harvested from a subset of dams (*n* = 3–4/group) and placenta was harvested from a subset of offspring (*n* = 2 fetuses/dam) for storage in RNAlater. At weaning, pancreas and liver were harvested from a subset of offspring and stored in RNAlater (*n* = 1/sex/litter/group, except for 2 litters with only females and 1 litter with only males; *n* = 7–10 litters). At endpoint (ie, 10 weeks post-HFD; postnatal weeks 22–27), islets were isolated from a subset of offspring and stored in RNAlater for qPCR analysis (*n* = 1/sex/litter; *n* = 4–6 litters/group); pancreas was harvested from a different subset of offspring for storage in 4% PFA for histological analysis (*n* = 1/sex/litter; *n* = 3–5 litters/group) (Figs. 4–8 and Supplementary Figs. 2 and 3).

#### Metabolic assessments

All metabolic analyses were performed in conscious, restrained mice, and blood samples were collected via the saphenous vein using heparinized microhematocrit tubes. Blood glucose levels were measured using a handheld glucometer (Lifescan; Burnaby; Canada).

Offspring body weight and blood glucose were measured weekly or 2×/week throughout the study following a 4-h morning fast, except at P1 and weaning timepoints, which were non-fasted. Body weight and blood glucose were measured in dams at P1 following a 4-h morning fast; fasted saphenous blood was collected to measure plasma insulin levels by ELISA (ALPCO, No. 80-INSMSU-E01, Salem, NH). Non-fasted offspring trunk and saphenous blood were collected at P1 and weaning, respectively, and fasted saphenous blood was collected at 4 weeks of age to measure plasma insulin levels by ELISA.

For all metabolic tests, time 0 indicates the blood sample collected prior to administration of glucose or insulin. For glucose tolerance tests (GTTs), mice received an i.p. bolus of glucose (2 g/kg: Figs. 5 and 7; 1 g/kg: Supplementary Figs. 3A and 3C) following a 4-h morning fast. Plasma samples were collected at 0, 15, and 30 min for measuring plasma insulin levels by ELISA. For insulin tolerance tests (ITTs), mice received an i.p. bolus of insulin (0.7 IU/kg) (Novolin ge Toronto; Novo Nordisk Canada, No. 02024233) following a 4-h morning fast.

#### EchoMRI analysis

Fat mass and lean mass were measured by the University of Ottawa Animal Behaviour Core (Ottawa, ON, Canada) at week 8 of the metabolic challenge using an EchoMRI-700 (EchoMRI LLC, Houston, TX). Percent fat and lean mass were determined relative to total body weight measured immediately prior to doing the MRI. The instrument was calibrated as per the manufacturer’s instructions prior to measurements.

#### Islet isolation

Islets were isolated from mice at 10 weeks post-HFD by pancreatic duct injection with collagenase (1000 units/ml; Sigma Aldrich, No. C7657) dissolved in Hanks’ balanced salt solution (HBSS: 137 mM NaCl, 5.4 mM KCl, 4.2 mM NaH_2_PO_4_, 4.1 mM KH_2_PO_4_, 10 mM HEPES, 1 mM MgCl_2_, 5 mM dextrose, pH 7.2). Pancreata were incubated at 37°C for 12 min, vigorously agitated, and the collagenase reaction quenched by adding cold HBSS with 1 mM CaCl_2_. Pancreas tissue was washed 3 times in HBSS+CaCl_2_ (centrifuging for 1 min at 180 rcf in between washes) and resuspended in Hams’s F-10 (Hyclone, No. SH30025.01, GE Healthcare Bio-Sciences, Pittsburgh, PA) containing 7.5% BSA and 1% penicillin–streptomycin (Corning, No. 30-002-CI, Tewksbury, MA). Pancreas tissue was filtered through a 70-μm cell strainer and islets were handpicked under a dissecting scope to >95% purity.

#### Quantitative real-time PCR

RNA was isolated from intact pancreas tissue, liver, and placenta stored in RNAlater using TRIzol (Invitrogen, No. 15596018, Carlsbad, CA), as per the manufacturer’s instructions. RNA was isolated from islets stored in RNAlater using the RNeasy Micro Kit (Qiagen, No. 74004, Hilden, Germany), as per the manufacturer’s instructions with the following amendment: 7 volumes of buffer RLT+β-ME were added to the samples prior to lysing with 70% ethanol. DNase treatment was performed prior to cDNA synthesis with the iScript gDNA Clear cDNA Synthesis Kit (Biorad, No. 1725035, Mississauga, ON, Canada). qPCR was performed using SsoAdvanced Universal SYBR Green Supermix (Biorad, No. 1725271) and run on a CFX384 (Biorad). *Hprt* or *PPIA* were used as the reference gene because these genes displayed stable expression under control and treatment conditions. Data were analyzed using the 2^−ΔΔCT^ relative quantitation method. Primer sequences are listed in Supplementary Table 1.

#### Immunofluorescent staining and image quantification

PFA-fixed pancreas tissues were processed and paraffin-embedded by the University of Ottawa Heart Institute Histology Core Facility (Ottawa, ON, Canada). Immunofluorescent staining was performed on tissue sections (5 µm thick), as previously described (Ibrahim *et al.*, 2020). The following primary antibodies were used: rabbit anti-insulin (1:200, C27C9; Cell Signaling Technology, No. 3014, Danvers, MA), mouse anti-insulin (1:250 L6B10; Cell Signaling, No. 8138S), mouse anti-glucagon (1:250, Sigma-Aldrich, No. G2654), rabbit anti-MAFA (1:1000; shared by Dr. Timothy Kieffer), and mouse anti-PCNA (1:100; BD Transduction Laboratories, No. 610665, San Jose, CA). The following secondary antibodies were used: goat anti-rabbit IgG (H + L) secondary antibody, Alexa Fluor 594 (1:1000; Invitrogen, No. A11037); and goat anti-mouse IgG (H + L) secondary antibody, Alexa Fluor 488 (1:1000; Invitrogen, No. A11029).

Apoptosis was assessed using the Molecular Probes Click-It Plus TUNEL Assay with Alexa Fluor 488 dye (Invitrogen, No. C10617), according to manufacturer’s instructions. Pancreas sections were counterstained with rabbit anti-insulin (1:200, C27C9; Cell Signaling Technology, No. 3014, Danvers, MA) and goat anti-rabbit IgG (H + L) secondary antibody, Alexa Fluor 594 (1:1000; Invitrogen, No. A11037) with no antigen retrieval, or with rat anti-CK19 (1:50; DSHB, TROMA-III-s) and goat anti-rat IgG (H + L) secondary antibody, Alexa Fluor 594 (1:1000; Invitrogen, No. A11007) with a 10-min heat-induced epitope retrieval. For every round of staining, a pancreas section was treated with DNase prior to TUNEL staining as a positive control.

For islet morphology quantification, a range of 2–17 islets were imaged per mouse with an Axio Observer 7 microscope and the average of all islet measurements is reported for each biological replicate. Immunofluorescence was manually quantified using Zen Blue 2.6 software (Carl Zeiss, Germany). The % hormone^+^ area per islet was calculated as [(hormone^+^ area/islet area)×100]. The % MAFA^+^ insulin^+^ cells per islet was calculated as [(number of MAFA^+^ insulin^+^ cells per islet)/(total number of insulin^+^ cells per islet)×100], with an average of 375 insulin^+^ cells counted per mouse. The % of PCNA^+^ insulin^+^ cells per pancreas section was calculated as [(number of PCNA^+^ insulin^+^ cells per pancreas section)/(total number of insulin^+^ cells per pancreas section)×100], with an average of 738 cells counted per pancreas section.

#### Quantification and statistical analysis

All statistics were performed using GraphPad Prism 8.4.2 (GraphPad Software Inc., La Jolla, CA). Specific statistical tests are indicated in figure legends. Sample sizes are described in the ‘Animals’ section, in figure legends, and in Supplementary Figure 1. For all analyses, *p* < .05 was considered statistically significant. Statistically significant outliers were detected by a Grubbs’ test with α = 0.05. All data was tested for normality using a Shapiro–Wilk test and for equal variance using either a Brown–Forsyth test (for one-way ANOVAs) or an *F* test (for unpaired *t* tests). Non-parametric statistics were used in cases where the data failed normality or equal variance tests. Parametric tests were used for all two-way ANOVAs, but normality and equal variance were tested on area under the curve values and by one-way ANOVAs. A two-way mixed-effect model (REML) ANOVA was used in cases where data points were missing due to random reasons. Data in line and bar graphs display mean±SEM. Individual data points on bar plots are always biological replicates (ie, different mice).

## Results

###  

#### Low-dose TCDD exposure during pregnancy induced Cyp1a1 expression in dam and offspring pancreas at P1

Dams were exposed to CO or 20 ng/kg/day TCDD 2×/week during mating and pregnancy, as previously described (Hoyeck *et al.*, 2020b); thus, offspring were indirectly exposed *in utero* via maternal exposure (Figure 1A). We were interested in whether maternally absorbed TCDD reached the developing offspring pancreas and used *Cyp1a1* and *Nqo1* as biomarkers of local AhR activation; liver was analyzed as a positive control because hepatic AhR activity is well-documented (Hoyeck *et al.*, 2020a; Ibrahim *et al.*, 2020; Matteo *et al.*, 2021). As expected, *Cyp1a1* was significantly upregulated in dam pancreas and liver mid-gestation, but surprisingly, the degree of induction in pancreas was more pronounced than in liver (Figure 1B). At P1, *Cyp1a1* was significantly upregulated in TCDD-exposed offspring pancreas and liver compared with controls (Figs. 1D and 1E), but was no longer upregulated in TCDD-exposed dam liver (Figure 1C) and only modestly increased in TCDD-exposed dam pancreas (3.5-fold increase at birth vs 17-fold increase at GD15.5; Figs. 1B and 1C). *Nqo1* was unchanged in TCDD-exposed dam (Figs. 1F and 1G) and female offspring tissues (Figure 1H), but was upregulated in TCDD-exposed male offspring liver and downregulated in male offspring pancreas at P1 (Figure 1I). Collectively, these data indicate that TCDD crosses the placenta and reaches the developing offspring pancreas and liver, and that AhR activation in dams appears to diminish over the course of pregnancy. These data also confirm that *Cyp1a1* is a more robust marker of dioxin exposure compared with other AhR target genes.

#### TCDD-exposed dams and offspring were hypoglycemic at P1

We next investigated whether *in utero* TCDD exposure disrupted metabolic health at birth. TCDD exposure did not affect litter size (CO: 8.03 ± 0.30 pups/litter; TCDD: 8.17 ± 0.39 pups/litter) or cause congenital abnormalities. There was also no change in dam body weight just prior to giving birth (Figure 2A). TCDD-exposed offspring had normal birth weight (Figs. 2D and 2G) but were hypoglycemic compared with controls (Figs. 2E and 2H), which corresponded with transient hypoglycemia in TCDD-exposed dams at P1 (Figure 2B). TCDD did not affect fasted plasma insulin levels in dams (Figure 2C) or random-fed plasma insulin in pups (Figs. 2F and 2I) at P1.

#### Low-dose TCDD exposure in utero reduced % β-cell area per islet in female but not male offspring at P1

We performed immunofluorescence staining of offspring pancreas at P1 to determine whether the observed hypoglycemia (Figs. 2E and 2H) coincided with changes in islet composition. *In utero* TCDD exposure significantly decreased % insulin^+^ area per islet in female (Figure 3B) but not male (Figure 3F) offspring. There was no change in average islet size (Figs. 3A and 3E), % glucagon^+^ area per islet (Figs. 3C and 3G), or % MAFA^+^ β-cells (Figs. 3D and 3H) in female or male offspring. Representative images of insulin, glucagon, and MAFA immunoreactivity are provided as examples (Figs. 3I and 3J). We found no TUNEL^+^ insulin^+^ or TUNEL^+^ CK19^+^ cells in any of the pancreas sections from either treatment group (representative images are provided for TUNEL staining, including a DNase-treated positive control section; Figs. 3K and 3L), so the decrease in β-cell area in female pups was not explained by increased β-cell or endocrine progenitor cell apoptosis at birth.

#### TCDD-exposed male offspring were transiently hypoglycemic at weaning, but no persistent effects on body weight, blood glucose, or plasma insulin levels were observed in either sex

Since reduced β-cell mass at birth could predispose offspring to metabolic disease later in life, we conducted a follow-up study to assess the long-term effects of early-life TCDD exposure on glucose homeostasis in male and female offspring. A new cohort of dams were injected with CO or 20 ng/kg/day TCDD 2×/week during mating, pregnancy, and lactation as previously described (Hoyeck *et al.*, 2020b); offspring were indirectly exposed to CO or TCDD via dams *in utero* and throughout lactation (Figure 4A). CO- and TCDD-exposed offspring were tracked until postnatal week 12–17 (‘Post-TCDD’ window), after which a subset were transferred to 45% HFD or remained on chow for an additional 10 weeks (‘Metabolic Challenge’ window; Figure 4A).

*Cyp1a1* was significantly upregulated in female (Figure 4B) and male (Figure 4I) offspring pancreas and liver at weaning, confirming that maternal TCDD continues to reach the developing offspring pancreas during lactation. At weaning, TCDD caused hypoglycemia in male offspring (Figure 4K) but did not affect random-fed blood glucose levels in female offspring (Figure 4D), body weight in either sex (Figs. 4C and 4J) or random-fed plasma insulin levels in either sex (Figs. 4E and 4L). There was also no lasting effect of early-life TCDD exposure on body weight (Figs. 4F and 4M), fasting blood glucose levels (Figs. 4G and 4N), or fasting plasma insulin levels (Figs. 4H and 4O) between postnatal weeks 4–17 in chow-fed offspring. Therefore, TCDD-induced hypoglycemia persisted longer in male than female offspring, but resolved in both sexes once TCDD exposure stopped at weaning.

#### TCDD-exposed male offspring showed transient glucose intolerance and reduced glucose-stimulated plasma insulin levels

Early-life TCDD exposure (until weaning) had no effect on glucose tolerance (Figs. 5A and 5D), glucose-stimulated plasma insulin levels (Figs. 5B and 5E), or insulin sensitivity (Figs. 5C and 5F) in chow-fed female offspring between postnatal weeks 5–17. Male offspring showed slight hyperglycemia at 30-min post-glucose at week 5–6 (Figure 5G) and reduced overall plasma insulin levels during the GTT at postnatal week 14–17 (Figure 5K—AUC), but these effects were modest and transient (Figs. 5G, 5H, 5J, and 5K). Early-life TCDD exposure had no effect on insulin sensitivity in male offspring (Figs. 5I and 5L).

#### Early-life TCDD exposure increased % fat mass in HFD-fed females but decreased % fat mass in HFD-fed males relative to chow-fed mice

We previously reported that TCDD exposure in adult female mice impairs metabolic adaptability to HFD feeding both during and post TCDD exposure (Matteo *et al.*, 2021; Hoyeck *et al.*, 2020b). As such, we were interested in whether early-life exposure to TCDD would impact metabolic health of offspring when challenged with HFD feeding later in life. Our data show that early-life TCDD exposure had no effect on body weight gain (Figs. 6A and 6E) or fasting glucose levels (Figs. 6B and 6F) following a HFD challenge in either sex. There was a trending decrease in % fat mass in TCDDChow females compared with COChow (Figure 6C) so we normalized % fat mass in HFD-fed mice relative to their chow-fed counterparts (ie, TCDDHFD/TCDDChow and COHFD/COChow). It was clear that TCDD-exposed female offspring gained more fat mass after HFD feeding than CO-exposed females (Figure 6D). Male offspring had the opposite trend, with TCDD-exposed males showing a less pronounced increase in fat mass than CO-exposed males after HFD feeding (Figs. 6G and 6H).

#### Early-life TCDD exposure delayed the onset of hyperglycemia and caused more pronounced hyperinsulinemia in HFD-fed female offspring

Consistent with the tracking data prior to HFD feeding (Figure 5), there continued be no long-term effect of early-life TCDD exposure on glucose tolerance (Figs. 7A, 7C, 7E, and 7G), glucose-stimulated plasma insulin levels (Figs. 7B, 7D, 7F, and 7H), or insulin sensitivity (Supplementary Figs. 2B and 2D) in chow-fed offspring of either sex (TCDDChow vs COChow).

After 1.5 weeks of HFD feeding, TCDD-exposed females were significantly less hyperglycemic than CO-exposed females (Figure 7A), although after 8–9 weeks of HFD feeding, COHFD and TCDDHFD females had similar levels of diet-induced hyperglycemia (Figure 7C and Supplementary Figure 2A). At 1.5 weeks post-HFD, both COHFD and TCDDHFD females displayed modest hyperinsulinemia at 30-min post-glucose compared with chow-fed females (Figure 7B), but after 8 weeks, only TCDDHFD females were significantly hyperinsulinemic compared with chow-fed females (Figure 7D). Insulin sensitivity of chow- and HFD-fed females was not affected by early-life TCDD exposure (Supplementary Figure 2B).

Male offspring were hyperglycemic (Figs. 7E and 7G and Supplementary Figure 2C) and hyperinsulinemic (Figs. 7F and 7H) after 1.5 weeks (Figs. 7E and 7F) and 8–9 weeks (Figs. 7G and 7H and Supplementary Figure 2C) of HFD feeding, irrespective of early-life TCDD exposure. The hyperinsulinemia in TCDDHFD males was initially more modest than COHFD males at 1.5 weeks post-HFD (Figure 7F), but by 8 weeks post-HFD, the degree of hyperinsulinemia was comparable between TCDDHFD and COHFD males (Figure 7H). Note that blood glucose levels in HFD-fed males exceeded the upper detection limit of our glucometer during the GTT at 8 weeks post-HFD (Figure 7G) so we repeated the GTT on HFD-fed males only with a lower dose of glucose and confirmed no differences in glucose intolerance (Supplementary Figure 2C). There were no differences in insulin sensitivity between any of the groups (Supplementary Figure 2D).

In summary, early-life TCDD exposure in females delayed the onset of hyperglycemia and led to more pronounced hyperinsulinemia following a transition to HFD feeding in adulthood. Whether this hyperinsulinemia in TCDDHFD females persists longer-term remains to be investigated. There were no lasting effects of early-life TCDD exposure on glucose homeostasis in male offspring.

#### TCDDChow female offspring continued to have reduced % β-cell area into adulthood and TCDDHFD females had increased % PCNA^+^ β-cells

Early-life TCDD exposure had no effect on average islet size (Figs. 8A and 8F) or % glucagon^+^ area per islet (Figs. 8D and 8I) in female and male mice fed a chow or HFD diet. Interestingly, TCDDChow females continued to display a significant decrease in % insulin^+^ area per islet compared with COChow females (Figure 8C) but no changes in % insulin^+^ area was observed in HFD-fed female or in male offspring (Figs. 8B, 8C, 8G, and 8H). TCDDHFD female offspring showed a significant increase in % PCNA^+^ insulin^+^ cells compared with all other groups (Figs. 8E and 8K); there were no significant differences in % PCNA^+^ insulin^+^ cells in male offspring (Figs. 8J and 8K).

Lastly, we assessed whether early-life TCDD exposure impacted islet hormone gene expression following HFD-feeding. HFD feeding caused a decrease in *Sst* (Supplementary Figure 3D) and *Ppy* (Supplementary Figure 3E) in female offspring islets, irrespective of chemical exposure, whereas *Ins1*, *Ins2*, and *Gcg* expression remained unchanged (Supplementary Figs. 3A–3C). There were no statistically significant differences in hormone gene expression in male offspring islets (Supplementary Figs. 3F–3J).

## Discussion

This study demonstrates that transient low-dose TCDD exposure during fetal and neonatal development in mice has minimal long-term metabolic health effects in chow-fed offspring, but alters metabolic adaptation to HFD feeding in female offspring during adulthood. We show that maternal TCDD reaches the developing offspring liver and pancreas, tissues that are critical for regulating metabolic homeostasis. Importantly, TCDD-exposed pups were hypoglycemic at birth, which coincided with reduced % β-cell area in female offspring. TCDD-exposed male offspring were also hypoglycemic at weaning and displayed modest, although transient, glucose intolerance and reduced glucose-stimulated plasma insulin levels shortly after TCDD exposure ceased. When challenged with HFD feeding during adulthood, TCDD- and CO-exposed male offspring developed comparable levels of hyperglycemia, whereas TCDD-exposed female offspring had delayed hyperglycemia, an exaggerated hyperinsulinemic response, increase % fat mass, and increased % PCNA^+^ β-cells compared with CO-exposed females. This is in line with our previous findings that females are more susceptible to TCDD-induced metabolic disruption compared with males, especially when challenged with a HFD (Hoyeck *et al.*, 2020a,b; Matteo *et al.*, 2021).

Given the importance of the fetal/neonatal period for pancreas development and establishing β-cell mass (Baeyens *et al.*, 2018; Dhawan *et al.*, 2007; Gregg *et al.*, 2012; Salinno *et al.*, 2019), we were not surprised to see adverse effects of fetal TCDD exposure on birth outcomes. TCDD-exposed male and female offspring were hypoglycemic at birth, which persisted in males until weaning. Birth marks an important period of fetal-neonatal metabolic adaptation as the source of nutrients from the dam to offspring is terminated and the offspring begin to rely on their own energy supply (Hawdon, 2016; Hussain *et al.*, 2015). Hypoglycemia at birth could be an early sign of impaired metabolic adaptability (Hawdon, 2016; Hussain *et al.*, 2015). Interestingly, this was accompanied by reduced % β-cell area in TCDD-exposed female but not male pups at birth. The decrease in % β-cell area was not explained by increased apoptosis of either β-cells or CK19^+^ ductal cells (the location of endocrine progenitor cells) at P1. Additionally, the proportion of β-cells expressing MAFA at P1 was not affected by TCDD exposure, suggesting that β-cell maturation occurred normally. The total endocrine area at P1 was also unaffected by TCDD, suggesting that the reduced β-cell area in female pups is driven by defects in β-cell lineage specification from endocrine progenitors, an earlier wave of β-cell death, and/or impaired β-cell expansion/proliferation during embryonic development. The impact of fetal TCDD exposure on β-cell growth and differentiation during late embryonic development should be further investigated.

We also detected a modestly reduced % β-cell area in TCDDChow compared with COChow females at 22–27 weeks of age (19–24 weeks since their last TCDD exposure), implying that the early loss of β-cells in females during fetal development was persistent. Interestingly, TCDDHFD females displayed increased β-cell proliferation, which could reflect an adaptive response to the pre-existing reduced β-cell area observed in TCDD-exposed female pups at birth. Reduced β-cell mass is well documented in individuals with Type 2 diabetes, and is believed to play an important role in disease progression (Ashcroft and Rorsman, 2012; Butler *et al.*, 2003; Cerf, 2013; Donath and Halban, 2004; Meier and Bonadonna, 2013; Ritzel *et al.*, 2006). However, some studies suggest that glucose intolerance only starts to manifest following a ∼50% reduction in beta cell mass (Butler *et al.*, 2003; Chen *et al.*, 2017; Dm *et al.*, 1990; Menge *et al.*, 2008). As such, the modest reduction in β-cell area observed in TCDD-exposed female offspring from our study was likely insufficient to induce glucose intolerance but would be expected to increase susceptibility to diabetes when faced with other metabolic challenges. Future studies should investigate the impact of introducing a metabolic challenge earlier in life (eg, at weaning) in TCDD-exposed offspring. This would further challenge the ability of female offspring to compensate for their reduced β-cell area, and also test whether the transient glucose intolerance and reduced plasma insulin observed in TCDD-exposed male offspring would have persisted or worsened with an earlier metabolic challenge. We would also encourage a follow-up study using oral TCDD exposure (ie, via food or gavage) that continues after weaning to better mimic chronic human exposure.

There were parallels between the metabolic outcomes in TCDD-exposed dams versus offspring, but the phenotypes in offspring were generally far more modest compared with dams (Hoyeck *et al.*, 2020b). TCDDHFD dams showed accelerated weight gain and pronounced hyperglycemia, dysregulated glucose-stimulated plasma insulin levels (initially hypoinsulinemia followed by prolonged hyperinsulinemia), reduced islet size, decreased MAFA^+^ β-cells, and increased cytoplasmic proinsulin accumulation compared with COHFD dams (Hoyeck *et al.*, 2020b). One interesting similarity between TCDDHFD dams and female offspring was an increase in % fat mass compared with COHFD, suggesting that low-dose TCDD exposure impairs energy storage and favors lipid accumulation in females. TCDD-exposed dams and female offspring also both eventually developed exaggerated HFD-induced hyperinsulinemia. Although TCDDHFD female offspring were not more glucose intolerant compared with COHFD female offspring, we speculate that ongoing exaggerated hyperinsulinemia in TCDDHFD females could eventually lead to β-cell exhaustion and worsened glucose intolerance compared with COHFD mice (Esser *et al.*, 2020). Longer-term studies are needed to support this hypothesis. Future studies should also assess insulin secretion in isolated islets *ex vivo* to determine whether β-cell dysfunction is driving the observed hyperinsulinemia *in vivo*.

The starkly different magnitude of metabolic abnormalities in dams versus offspring could be due to the different modes of TCDD exposure (ie, direct vs indirect), leading to different levels of TCDD accumulation. Human pregnancy studies show that most POP congeners are more abundant in maternal blood and breast milk compared with offspring cord blood (Meijer *et al.*, 2008; Nakamura *et al.*, 2008; Needham *et al.*, 2011), with a transfer ratio of 0.5–1 between mother and offspring (Meijer *et al.*, 2008). However, some POP congeners have been found at similar or even higher concentrations in offspring tissues compared with maternal serum/breast milk (Meijer *et al.*, 2008; Nakamura *et al.*, 2008; Needham *et al.*, 2011). Our study showed that TCDD exposure induced a more pronounced fold-induction of *Cyp1a1* expression in offspring pancreas and liver than dam tissues at P1. We also saw a significant upregulation of *Nqo1*, another AhR gene target, in liver from TCDD-exposed male offspring but not dams or female pups at P1. Collectively, this could reflect greater TCDD accumulation and/or a more sensitive AhR pathway response in developing offspring compared with dams. Future studies should compare TCDD concentration in maternal and offspring pancreas. Differences in long-term metabolic consequences of TCDD exposure on dams versus offspring could also reflect changes in β-cell plasticity during adulthood compared with fetal/neonatal stage of development. Although early life marks an important period for β-cell development, β-cells display more plasticity during this period compared with adulthood, potentially allowing younger β-cells to recover from any detrimental effects caused by transient TCDD exposure (Remacle *et al.*, 2007).

Our data is in line with previous findings showing increase susceptibility of female mice to TCDD-induced metabolic defects compared with males (Hoyeck *et al.*, 2020b; Matteo *et al.*, 2021). Interestingly, some studies have shown cross-talk between the AhR and estrogen signaling pathways (Gargaro *et al.*, 2021; Matthews and Gustafsson, 2006). Estrogen plays an important role in maintaining normal body composition and glucose homeostasis (Tramunt *et al.*, 2020). As such, AhR activation following TCDD exposure could interfere with the protective effects of estrogen and disrupt metabolic homeostasis in females. However, there are still significant gaps in our understanding of sex-specific effects of TCDD, and other persistent organic pollutants, on metabolic health. Studies in AhR knockout mouse models will help to better elucidate the role of AhR in driving sex-specific metabolic effects of TCDD.

Collectively, our study indicates that maternally absorbed TCDD reaches the developing offspring pancreas and has modest sex-specific effects on glycemia and islet composition in offspring. TCDD-exposed female, but not male, offspring had delayed onset of HFD-induced hyperglycemia compared with controls, but eventually developed exaggerated hyperinsulinemia, which could be detrimental if persistent. Additionally, our study suggests that even transient low-dose exposure to pollutants during development has persistent effects on % β cell area in female offspring. Our findings add to a growing body of literature emphasizing the importance of considering sex when investigating the effect of pollutants on diabetes risk.

## Declaration of conflicting interests

The author/authors declared no potential conflicts of interest with respect to the research, authorship, and/or publication of this article.

## Supplementary Material

kfad042_Supplementary_Data
