# Supplementary material for: Fetal and neonatal dioxin exposure causes sex-specific metabolic alterations in mice
Source: Toxicol Sci. 2023 Apr 28;194(1):70–83. doi: 10.1093/toxsci/kfad042 (PMC10306400; doi:10.1093/toxsci/kfad042)
Supplement: kfad042_Supplementary_Data [file kfad042_supplementary_data.docx]

**Fetal and neonatal dioxin exposure causes sex-specific metabolic alterations in mice**

Myriam P Hoyeck^1^, Rayanna C Merhi^1^, Cameron Tulloch^1^, Kaitlyn McCormick^1^, Shahen Mohammed Abu Hossain^1^, Antonio A Hanson^1^, Jennifer E Bruin^1^*

^1^Department of Biology & Institute of Biochemistry, Carleton University, Ottawa, Ontario, K1S 5B6 Canada.

* Address correspondence to:

Dr. Jennifer Bruin

1125 Colonel By Drive

Ottawa, ON K1S 5B6

T: 613-520-2600 x3656

jenny.bruin@carleton.ca

**Supplementary Table 1:** Primer sequences for qPCR.

| **Target** | **Forward Sequence (5’-3’)** | **Reverse Sequence (5’-3’)** | **Amplicon Size (bp)** | **Amplification Efficiency (%)** |
| --- | --- | --- | --- | --- |
| *Hprt* | GCT GAC CTG CTG GAT TAC AT | TTG GGG CTG TAC TGC TTA AC | 242 | 122.4 |
| *PPIA* | GCC AGG ACC TGT ATG CTT TA | AGC TCT GAG CAC TGG AGA GA | 178 | 102 |
| *Cyp1a1* | ATC ACA GAC AGC CTC ATT GAG C | AGA TAG CAG TTG TGA CTG TGT C | 139 | 135.0 |
| *Nqo1* | CTC TGG CCG ATT CAG AGT GG | GTC TCC TCC CAG ACG GTT TC | 152 | 119 |
| *Ins1* | TCA GAG ACC ATC AGC AAG CA | CTC CCA GAG GGC AAG CAG | 89 | 105 |
| *Ins2* | GCT TCT TCT ACA CAC CCA TGT | ACG ACT GAT CTA CAA TGC CAC | 147 | 98 |
| *Gcg* | ACT CAC AGG GCA CAT TCA CC | CCA GTT TAT AAA GTC CCT GG | 353 | 100 |
| *Sst* | CTG AGC AGG ACG AGA TGA GG | TAA CAG GAT GTG AAT GTC TTC CAG AA | 121 | 100 |
| *Ppy* | CGC ATA CTG CTG CCT CTC C | CCT GGT CAG TGT GTT GAT GTA TCT G | 178 | 105 |

**
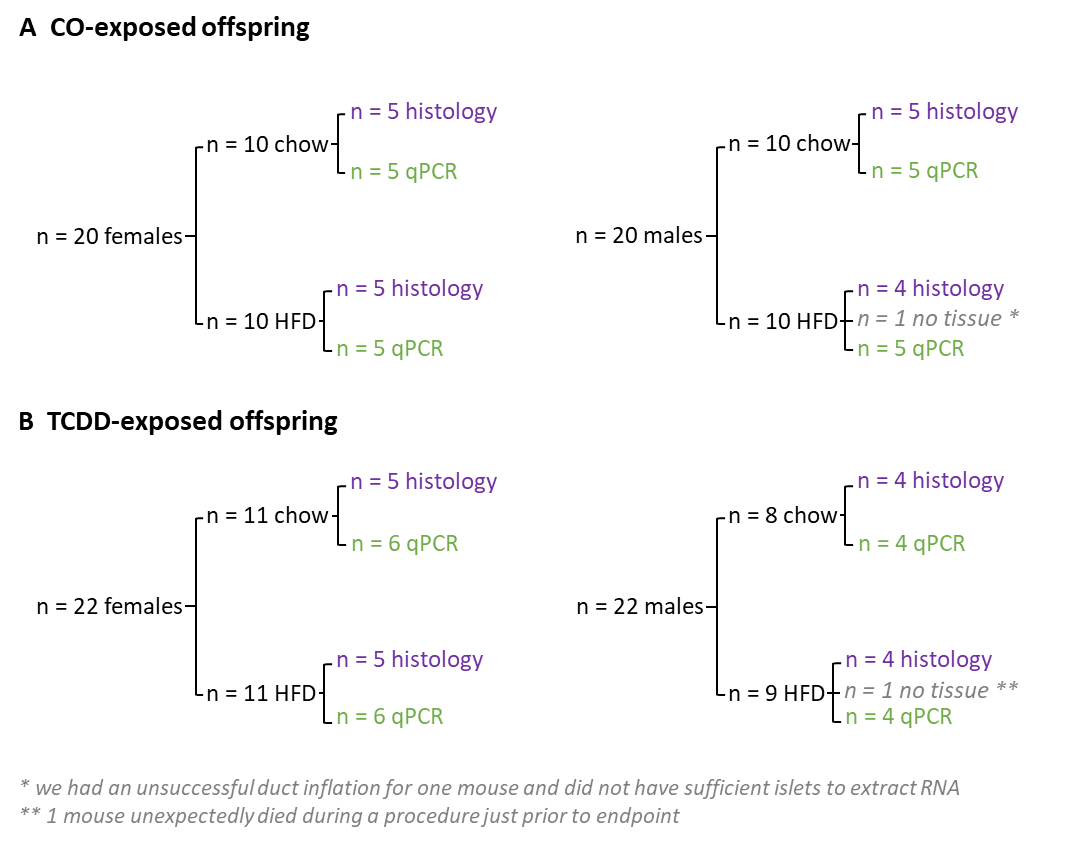
**

**Supplementary Figure 1: A breakdown of the sample size used in cohort 2.** We generated **(A)** 20 CO-exposed and **(B)** 22 TCDD-exposed offspring per sex; these offspring were generated from 10 different litters, with ~3 offspring/litter/sex. At postnatal week 12-17, offspring were transferred to chow or high-fat diet (HFD) feeding (n = 8-11/sex/group), and were used for either histological analysis (n = 4-5/experimental group; n=1/sex/litter) or islet isolations for qPCR analysis (n = 4-6/experimental group; n=1/sex/litter).

**
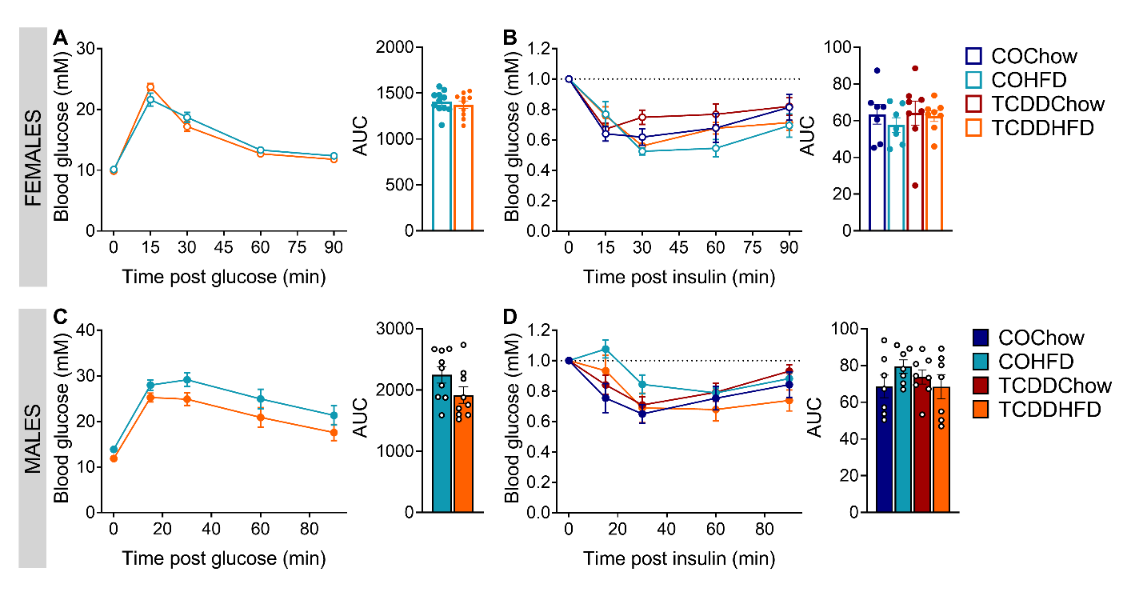
**

**Supplementary Figure 2: Early-life exposure to TCDD did not have long-term effects on glucose tolerance and insulin sensitivity in chow or HFD-fed offspring. (A,C)** A glucose and **(B,D)** insulin tolerance test was performed at week 9 of the metabolic challenge on **(A,B)** female and **(C,D)** male offspring (n = 1-2/sex/litter, n = 8-10 different litters/group). The glucose tolerance test was performed on HFD-fed male and female offspring only using a glucose dose of 1 g/kg. All data are presented as mean ± SEM. Individual data points in bar graphs represent biological replicates (different mice). The following statistical tests were used: **(A,C)** line graph, two-way RM ANOVA with Sidak’s multiple comparison test; bar graph, two-tailed unpaired t-test, **(B,D)** line graph, two-way REML ANOVA with Tukey’s multiple comparison test, **(A-D)** bar graph, two-way ANOVA with Tukey’s multiple comparison test.


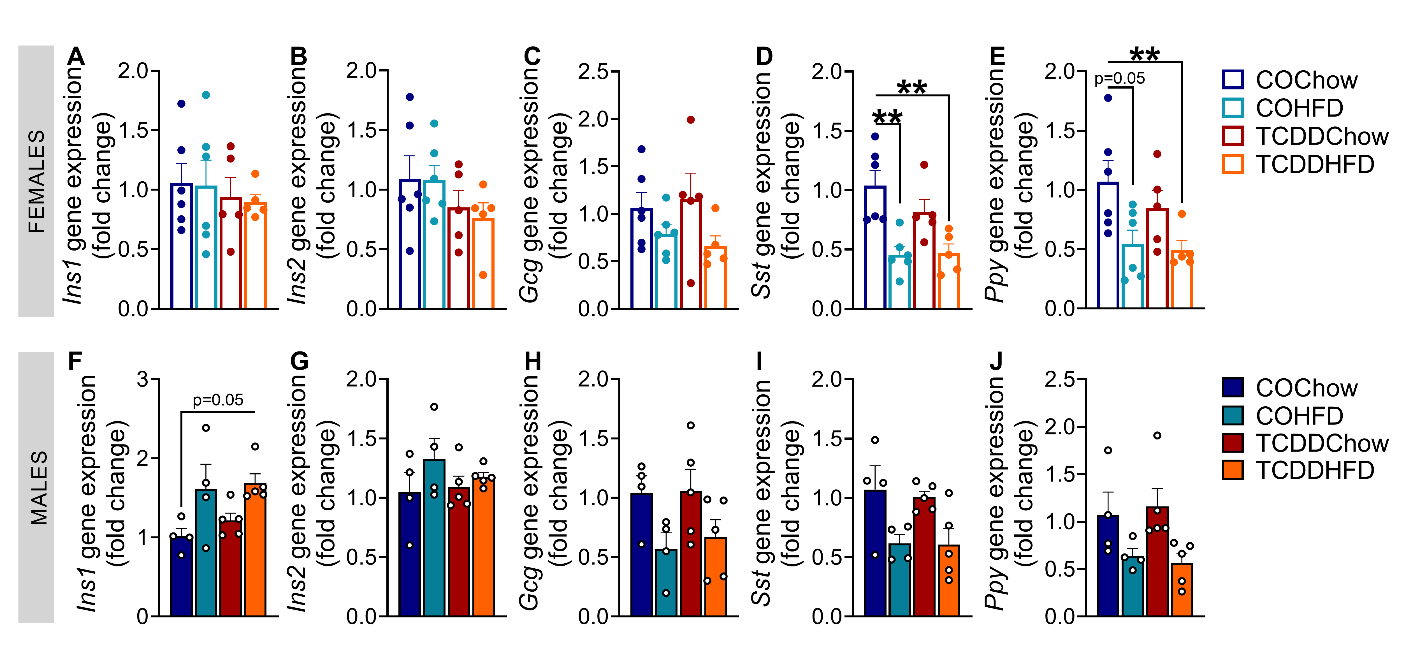
**Supplementary Figure 3: Early-life TCDD-exposure did not alter islet hormone gene expression in chow or HFD-fed offspring.** Islets were isolated from offspring at 10 weeks post-HFD for qPCR analysis (see Figure 4A for study timeline) (n = 1/sex/litter, n = 4-6 different litters/experimental group). **(A,F)** *Insulin-1 (Ins1)*, **(B,G)** *insulin-2* *(Ins2)*, **(C,H)** *glucagon (Gcg)*, **(D,I)** *somatostatin (Sst)*, and **(E,J)** *pancreatic polypeptide (Ppy)* expression were measured in **(A-E)** female and **(F-J)** male offspring. All data are presented as mean ± SEM. Individual data points in bar graphs represent biological replicates (different mice). The following statistical tests were used: **(A-J)** two-way ANOVA with Tukey's multiple comparison test.
